# Effects of Composite Warm Mix Additive (CAR) on the Physical and Rheological Performance of Bitumen and the Pavement Performance of Its Concrete

**DOI:** 10.3390/ma12233916

**Published:** 2019-11-27

**Authors:** Jizhe Zhang, Peizhao Li, Changjun Sun, Ming Liang, Yuanyuan Li, Zhanyong Yao, Xiaomeng Zhang

**Affiliations:** 1 School of Qilu Transportation, Shandong University, Jinan 250061, China; jizhe.zhang@sdu.edu.cn (J.Z.); 201814689@mail.sdu.edu.cn (P.L.); changjunsun2015@163.com (C.S.); ming.liang@sdu.edu.cn (M.L.); zhanyong-y@sdu.edu.cn (Z.Y.); 2 Nottingham Transportation Engineering Centre, Department of Civil Engineering, University of Nottingham, University Park, Nottingham NG7 2RD, UK; 3 Shandong Transportation Institute, Jinan 250031, China; zhangxiaomeng@sdjtky.cn

**Keywords:** warm mix bitumen, modification of bitumen, physical performance, rheological performance, pavement performance, bituminous concrete

## Abstract

Warm mix asphalt received increasing attention in recent years, and this technology aims to increase the fluidity of bitumen in the process of mixing and construction. To characterize the physical and rheological properties of bitumen and the pavement performance of bituminous mixtures, it was modified by a composite additive Rediset. Rediset consists of both the cationic surfactants and organic additive-based rheology modifiers. Commonly used materials such as Pen 60/80 bitumen and bituminous concrete (AC-20) were selected. The results show that Rediset can improve the penetration and softening point of the bitumen, making the bitumen stiffer and harder. All Rediset-modified bituminous concretes are in the same low-temperature performance grade (PG) as the bitumen without Rediset. Although Rediset can decrease the rutting and crack resistance of Rediset-modified bituminous concrete, all the Rediset-modified bituminous concrete with less than 2% Rediset still satisfied the requirement of the maximum bending strain being higher than 2000 με, and the dynamic stability of Rediset-modified bituminous concrete with 3% Rediset was still higher than 1000 cycles/mm. The cationic surfactants in the Rediset can play the role of an anti-stripping agent and improve the adhesion between the interfaces of the aggregate and bituminous binder, which enhances the moisture resistance of Rediset-modified bituminous concrete.

## 1. Introduction

Hot mix asphalt (HMA) is a conventional pavement material composed of bituminous binder, aggregate particles, and mineral filler, which is widely used in pavements worldwide [1]. As HMA is generally produced at high temperatures (140–190 °C), a large amount of fossil fuel is needed to heat the aggregates and bitumen up to required temperatures [2]. This, therefore, results in increased greenhouse gas (GHG) emission during the heating process and finally causes environmental pollution [3,4]. In order to prepare sustainable paving materials [5], different technologies were developed in the pavement industry with the view of reducing the consumption of non-recoverable fuel [6,7,8,9,10]. Recently, one of the plausible approaches of using warm mix asphalt (WMA) was developed to enhance the workability and fluidity of bituminous concrete during the mixing and paving process, allowing it to be constructed at a relatively lower temperature [11].

In comparison, the manufacturing temperature of WMA is in the range of 20–40 °C lower than that of HMA. The application of this technique confers many sustainable advantages to the asphalt pavement industry. Firstly, decreasing the producing temperature can reduce the consumption of non-renewable energy resources, which in turn minimizes the emission of GHG and other hazardous gases (e.g., aerosol and polycyclic aromatic hydrocarbons) [3]. It was reported that the energy consumption in the production of WMA is around 60–80% of that of HMA [12]. Secondly, the end product with a decreased production temperature can increase the haul distance and enable the bituminous pavement to be constructed during the cold season, which can also decrease the aging (due to the oxidation and volatilization in construction process) of bituminous binder [13]. Thirdly, the lower-temperature production of the asphalt mixture can provide safer working conditions for constructors, and the pavement can be opened earlier after the construction [14]. Furthermore, the WMA technique may reduce the viscosity of bituminous binders, which in turn improves the workability and the compatibility of bituminous concretes [15].

The principle of the WMA technique is to reduce the bitumen viscosity during mixing and compaction processes by adding appropriate additives [16]. These additives can be briefly divided into three categories: bitumen foaming, organic additives, and chemical additives [17,18]. However, there are still some disadvantages when using the chemical additives individually. The chemical modifiers generally include a combination of surfactants, emulsification additives, and anti-stripping components, which can reduce the tension and surface friction between aggregates and bituminous binders [19]. It was highlighted that the rheological performances of bitumen cannot be significantly improved by chemical WMA additives [20]. Furthermore, researchers also found that the bitumen viscosity remains almost unchanged after the addition of chemical additives [21]. We know that the lubricity can be used to characterize the internal friction coefficient of the bitumen, and it can be tested using the developed fixture in the dynamic shear rheometer (DSR), which is normally used to assess the lubricating effects of warm mix additives [22,23]. It was demonstrated that the workability improvement of bituminous mixtures is not only attributed to the increase of lubricity, but also to the reduction in friction between aggregates during compaction [24].

Based on this situation, the composite WMA additive would, therefore, be more interesting to reduce the viscosity of bitumen and increase lubricity between aggregates, which can be composed of cationic surfactants, as well as organic additive-based rheology modifiers. It was reported that the addition of such composite WMA additives, such as the composite additive Rediset (CAR), is able to achieve a temperature reduction of 15–30 °C [25]. Unlike other chemical additives, this modifier comprises a long-chain aliphatic hydrocarbon and a $-{\mathrm{NH}}_{3}^{+}$ group, which can improve the adhesive property of the aggregate–bitumen interface, resulting in better moisture resistance [26]. As the melting point of CAR is in the range of 80–90 °C, it can easily dissolve in the hot bitumen automatically without high-shear mixing. The influence of the composite WMA additive on the pavement performance of asphalt mixture was investigated widely. Bennert found that the permanent deformation of an asphalt mixture specimen prepared with 2% commercial CAR was lower than that containing 1% CAR [27]. In contrast, Elseifi reported that the rutting resistance of mixtures with the incorporation of CAR was lower than that of HMA [28]. A study conducted by Sampath showed that the asphalt mixtures containing CAR showed higher tensile strength ratio (TSR) than that prepared with Sasobit, and all of those values were lower than 80% [29].

It can be found that previous researches investigated the feasibility of such an additive used for asphalt mixtures. However, there is still limited investigation on the effect of the composite additive on the thermal cracking performance of bituminous mixtures. Moreover, previous investigations did not give consistent results [30]. Based on this situation, the objectives of this paper, involving the bituminous binder and the bituminous concrete were (a) to prepare the composite additive modified bitumen (CAMB) via the wet modification method, investigating the CAR and its dosage on the macro-performance (such as the physical performance and rheological performance) of bituminous binder, and (b) to determine the mix design of the CAMB concrete, investigating the CAR and its dosage on the anti-rutting, thermal crack resistance, and moisture sensitivity of the CAMB concrete.

In this research, firstly, the base bitumen was modified by adding CAR with different dosages (0 wt.%, 1 wt.%, 2 wt.%, and 3 wt.%). The effect of CAR on the physical performance of CAMB was investigated using both penetration and softening point parameters. Then, the bending-beam rheometer (BBR) test and Brookfield viscosity test were performed to characterize the rheological properties of CAMB. After that, a commonly used bituminous concrete with a nominal maximum aggregate size (NMAS) of 19 mm (AC-20) was designated with the CAMB, which was used to study the effect of CAR on the pavement performance of bituminous concrete, including rutting resistance, thermal cracking, and moisture sensitivity. The experimental results obtained from this paper can contribute to verifying the feasibility of CAR as a WMA additive for bitumen and bituminous concrete. In addition, the effect of CAR dosage on the technical performance of the bitumen and related concrete was characterized.

## 2. Materials and Experimental Methods

### 2.1. Materials

#### 2.1.1. Bituminous Binder

The 70# bitumen (Pen 60/80) was used as the neat bituminous binder, the technical performance of which is listed in Table 1. This bitumen is offered by Sinopec Qilu Petrochemical Company. The saturate, aromatic, resin, and asphaltene ratios of the bitumen were 16.3%, 56.7%, 8.8%, and 18.1%, respectively.

#### 2.1.2. Aggregates and Mineral Filler

The aggregate used was crushed limestone aggregate, and the filler used in the bituminous concrete was limestone powder. The physical properties of the aggregate and mineral filler were tested according to the criterion of Test Method of Aggregate for Highway Engineering (JTG E42-2005). The related parameters are shown in Table 2.

#### 2.1.3. Preparation of Composite Additive Modified Bitumen (CAMB)

A composite WMA additive CAR (WMA-8017) was used to modify the properties of binder and bituminous mixture, and it was offered by the AkzoNoble Surface Chemistry AB Sweden [30]. As a composite additive, it is composed of both surface-active components and organic agents. The surface-active components can act as anti-stripping agents and improve the adhesive performance of the aggregate–bitumen interface. Due to its low melting point, the addition of CAR to the base bitumen can decrease the viscosity of the bitumen, which in turn decreases the mixing and compaction temperature of the bituminous mixture during the construction process. Moreover, the CAR can develop a solid form in the bitumen and decrease the temperature sensitivity of bitumen. The physical and chemical information of Rediset is shown in Table 3.

The composite additive modified bitumen (CAMB) samples were produced using the following procedure: the base bitumen was firstly pre-heated to 150 °C for 1 h to make it soft enough to blend with the CAR. Then, the predetermined dosage (1%, 2%, and 3%) of CAR was added to the hot bitumen. The bitumen/CAR was conditioned at 150 °C for 10 min to melt the additive. Finally, the bitumen and the CAR were mixed using a high-speed mixer with a shearing speed of 2500 rpm for 30 min.

### 2.2. Experimental Methods

#### 2.2.1. Mix Design of Bituminous Mixture

**Figure 1:**
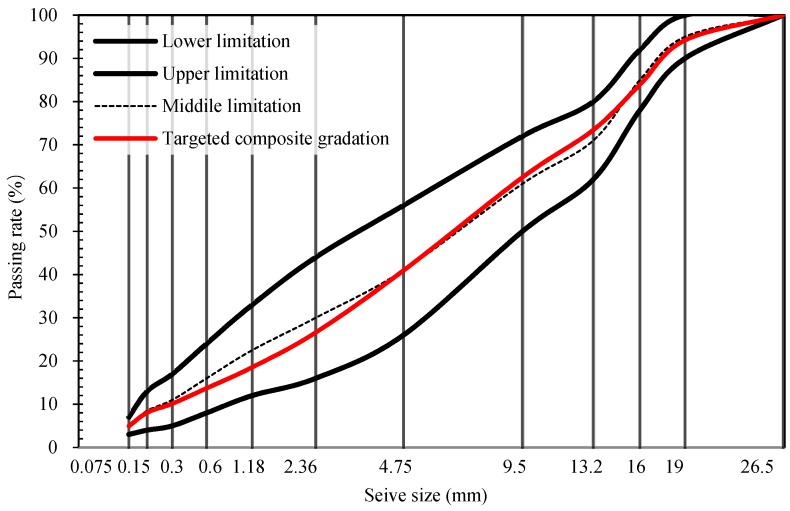
Composite aggregate gradation of AC-20 bituminous concrete.

A dense gradation asphalt mixture with an NMAS of 19 mm (AC-20) was used. the mixing ratio of the bituminous concrete was verified by the Marshall method (JTG E20-2011). The composite aggregate gradation is shown in Figure 1. The optimum binder dosage of the mixture was 4.3%, while the mixing temperature and the compaction temperature of the HMA mixture were 135 °C and 125 °C, respectively. This was about 30 °C lower than that of the HMA mixture.

#### 2.2.2. Physical Performance Tests of Bitumen

The ring-and-ball test (ASTM D36) was conducted to evaluate the softening point of bituminous binders before and after incorporating the composite additive. The penetration test (ASTM D5) was used to assess the effect of the composite additive on the bitumen hardening.

The penetration index (PI) of bituminous binders was then calculated using the softening point and penetration values according to Equation (1).

(1) $$\mathrm{PI}=\frac{1520-500\log\left(Pen_{25}\right)-20SP}{50\log\left(Pen_{25}\right)-SP-120},$$

where $Pen_{25}$ is the penetration at 25 °C (0.1 mm), and SP is the softening point (°C).

#### 2.2.3. Bending-Beam Rheometer (BBR) Test

The BBR test was carried out to assess the stiffness and creep performance of bituminous binders at low temperatures. Bitumen specimens with specific dimensions (127 × 12.7 × 6.35 mm) were firstly prepared and then conditioned at a predetermined temperature for at least 60 min. During the BBR testing, a constant loading (980 ± 50 mN) was imposed on the specimen, and the deformation was measured at the same time. The test was conducted at −12 °C, −18 °C, and −24 °C. The creep stiffness (S) and m value (m) could then be calculated at the constant loading time of 60 s [31]. S represents the bitumen’s resistance to a constant loading, while m represents the development rate of bitumen stiffness during the loading process. In an effort to guarantee appropriate cracking resistance behavior at low temperatures, the bitumen should avoid high tensile stress and ensure quick stress relaxation [32]. Based on the Superpave TM standard (American Association of State Highway and Transportation Official, AASHTO M 320), the S value should be not more than 300 MPa, while the m value should be higher than 0.300.

#### 2.2.4. Brookfield Viscosity Test

The Brookfield viscometer (ASTM D4402) was used to assess the influence of CAR on the viscosity of base bitumen. The rotational viscosity can not only estimate the fluidity of bitumen, but also reflect the compatibility between the bitumen and aggregate [33]. This test was performed with the temperature ranging from 105 °C to 165 °C.

#### 2.2.5. Wheel Tracking Test

The wheel tracking test (WTT) was performed to characterize the rutting resistance of asphalt mixtures before and after adding CAR. As the stress state induced in an asphalt slab is similar to that of the actual axial loading, the wheel tracking test is recognized as a reasonable approach for rutting assessment [34]. WTT slabs with dimensions of 300 mm × 300 mm × 50 mm were compacted in steel molds with a roller compactor. Before testing, they were conditioned at a predetermined testing temperature for at least 5 h to obtain homogeneous temperature distribution. During testing, the contact pressure of 0.7 MPa was set between the rubber tire and slab surface to simulate the traffic loading. A higher dynamic stability value can indicate better permanent deformation resistance.

#### 2.2.6. Three-Point Bending (3PB) Test

**Figure 2:**
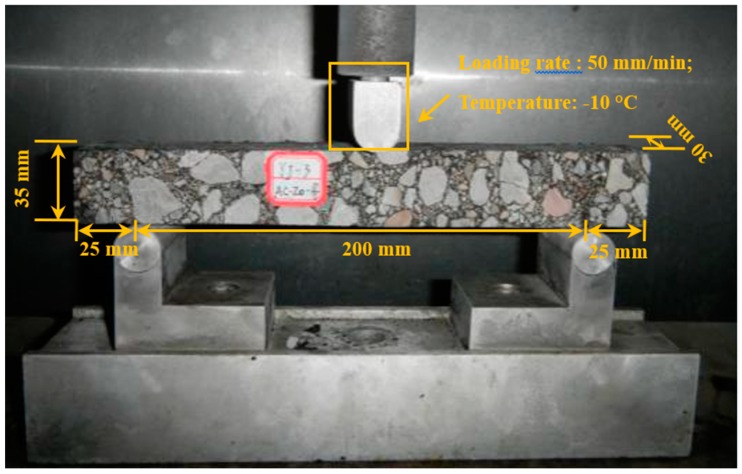
Three-point bending test of bituminous concrete beams.

The three-point bending (3PB) test (JTG E20-2011) was applied to assess the thermal cracking resistance of CAMB concrete. The dimension of the prismatic beam specimens was 250 × 30 × 35 mm. These asphalt specimens were firstly conditioned at −10 °C for 1.5 h. After that, the specimen was placed on two rollers with a span length of 200 mm. During testing, a concentrated loading with the displacement of 50 mm/min was applied in the mid-span of the specimen, as shown in Figure 2. Four replicates were performed on each asphalt mixture. The bending tensile strength ($R_{B}$), maximum bending strain ($\varepsilon_{B}$), and bending stiffness ($S_{B}$) could be calculated as shown in Equations (2)–(4).

(2) $$R_{B}=\frac{3LP_{P}}{2bh^{2}},$$

(3) $$\;\varepsilon_{B}=\frac{6hd}{L^{2}},\;$$

(4) $$\;S_{B}=\frac{R_{B}}{\varepsilon_{B}},\;$$

where h and b are the height and width of the specimen, respectively (mm), L is the distance between the two support rollers (mm), d is the deflection of the specimen (mm), and $P_{P}$ is the maximum loading (N).

#### 2.2.7. Indirect Tensile Test (ITT) and Moisture Sensitivity Test

The ITT test (JTG T0716-2011) was conducted to study the indirect tensile strength (ITS) of CAMB concrete with the results correlated to the rutting and cracking resistance of asphalt pavement [35]. Cylindrical specimens with a thickness of 63.5 ± 1.3 mm and diameter of 101.6 mm were prepared using a Marshall compacter. During testing, a uniform compressive loading speed of 50 mm/min was applied at the temperature of 25 °C. The maximum compressive loading was then recorded by the testing machine. Four replicates tests were performed on each asphalt mixture. The ITS was calculated according to Equation (5).

(5) $$\mathrm{ITS}=\frac{2P_{max}}{\pi td},$$

where $P_{max}$ is the maximum applied load (kN), and t and d are the thickness and diameter of the specimen, respectively (mm).

The moisture resistance of CAMB concrete was assessed by the ITS test before and after freeze–thaw cycles. Before testing, Marshall specimens were firstly vacuum-saturated and then subjected to a freeze–thaw cycle, in which the specimen was firstly frozen at −18 °C for 16 h and then soaked at 60 °C in a water bath for 24 h. The conditioned specimens were then immersed in a water bath for 2 h at 25 °C. Finally, the tensile strength ratio (TSR) was calculated according to Equation (6).

(6) $$\mathrm{TSR}=\frac{TS_{1}}{TS_{0}}\times 100\%,$$

where $TS_{0}$ and $TS_{1}$ are the ITS of CAMB concrete before and after freezing conditions, respectively (MPa).

## 3. Results and Discussion

### 3.1. Physical Properties of CAMB

The physical properties, namely, softening point (SP) and penetration of bituminous binders with the composite additive, are listed in Table 4. From Table 3, the SP value of bituminous binder increased with increasing CAR content; the SP values of 1% CAR-, 2% CAR-, and 3% CAR-modified bitumen were 0.2, 1.6, and 3.3 °C higher than that of the bitumen without CAR. In addition, the penetration of bitumen decreased gradually with increasing CAR dosage, whereby the penetration of CAMB with 3% CAR was 6.9% lower than that of bitumen without CAR. The results indicate that the CAR can enhance the high-temperature stability performance of the bituminous binder. Even though the CAR has a lower melting point than neat bitumen, it can present a solid form at in-service temperatures and contribute to the increased stiffness.

**Figure 3:**
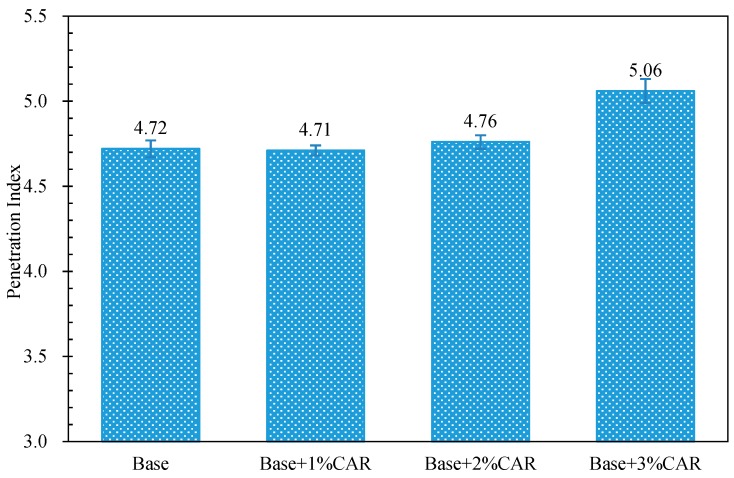
Penetration index (PI) values of composite additive modified bitumen (CAMB).

The penetration index (PI) of all bitumen samples are shown in Figure 3; it was calculated according to Equation (1). From Figure 3, the 1% CAR showed no obvious effect on the PI value of CAMB; with the further increase in CAR content (higher than 2%), the PI value increased gradually. For instance, the CAMB with 3% composite additive had the highest PI value, which was 7% higher than that of base bitumen. A higher PI value presents more elastic behavior and less temperature susceptibility [36]. Therefore, the CAR is able to enhance the elasticity of bitumen, as well as decrease its temperature susceptibility.

### 3.2. Rheological Performance of CAMB

#### 3.2.1. Creep Stiffness of CAMB

**Figure 4:**
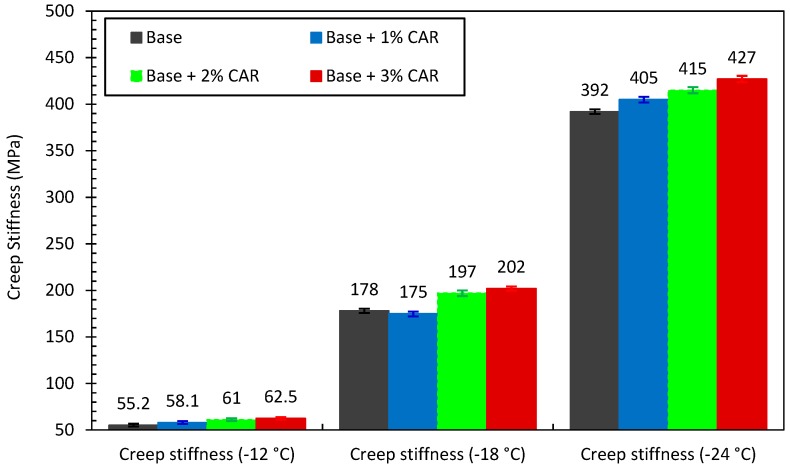
Creep stiffness of bitumen with and without CAR.

**Figure 5:**
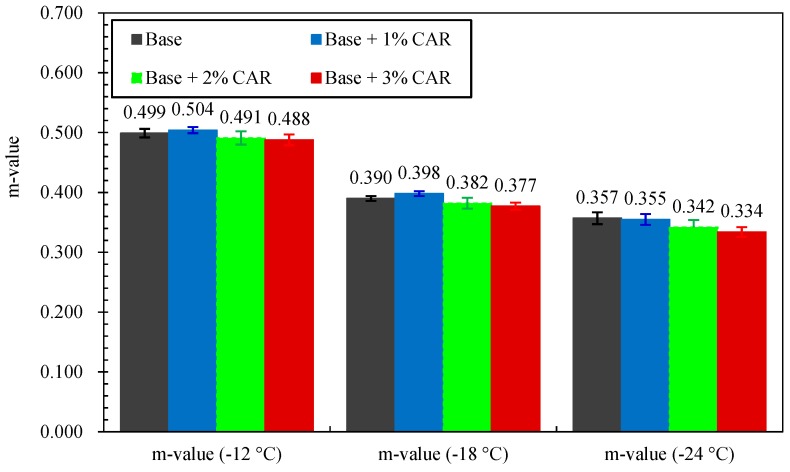
The m values of bitumen with and without CAR.

The creep stiffness (S) and m value (m) of the CAMB are shown in Figure 4 and Figure 5, respectively. As shown in Figure 4, for the same bitumen sample, the S value experienced an obvious increase as the temperature decreased from −12 °C to −24 °C. When the temperature remained constant, the addition of CAR resulted in an increase in S value. In contrast, the m value showed a decreasing trend with the decline in testing temperature. In addition, the increase in CAR dosage also resulted in a decrease in m value. This phenomenon indicated that the CAR somehow reduces the flexibility and the crack resistance of bitumen at low temperature.

During its service life, the asphalt pavement should have enough relaxation and avoid high tensile stress so as to resist thermal cracking at low in-service temperatures [37]. Based on this situation, the Superpave standard (AASHTO M 320) requires that the S value should be less than 300 MPa, while the m value should be greater than 0.300. According to this standard, the temperature when S = 300 MPa or m = 0.300 is defined as the failure temperature of related bitumen [38]. The failure temperature can be used to quantificationally evaluate the low-temperature crack resistance of bitumen. The lower the failure temperature is, the better the crack resistance of bitumen will be. The failure temperatures of bitumen were obtained based on curve fitting with the results shown in Table 5. It can be seen that all four of the binders obtained failure temperatures lower than −20 °C. The addition of CAR resulted in a slight increase in terms of failure temperature, which indicated that the low-temperature crack resistance of bitumen deteriorated. However, it should be mentioned that all CAMB samples were still in the same low-temperature PG grade (PG-28) as that of the base bitumen.

#### 3.2.2. Viscosity of CAMB

**Figure 6:**
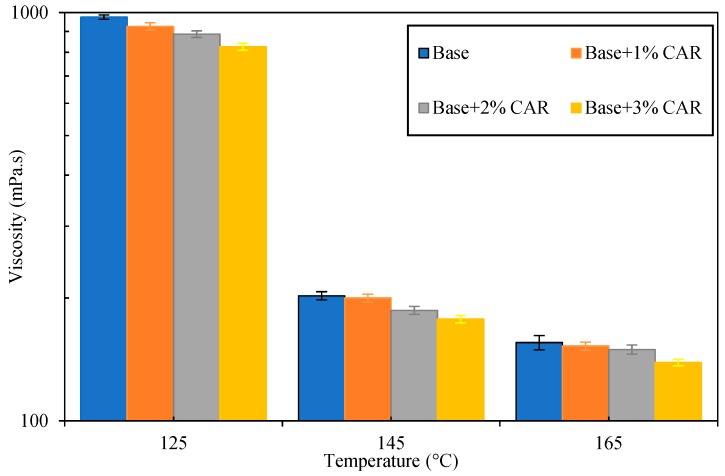
Viscosity of bituminous binders with or without CAR from 125 °C to 165 °C.

Normally, the construction temperatures for HMA is in the temperature range of 125–165 °C; the bitumen fluidity is very important to assess the blending efficiency and compaction quality of a bituminous mixture [39]. Thus, the viscosities of base bitumen and CAMB were measured using a rotational viscometer in the temperature range of 125–165 °C with the results shown in Figure 6. As shown in this figure, the base bitumen had the highest viscosity, while the addition of CAR resulted in a slight reduction in terms of the bitumen viscosity. The viscosity–temperature curve of CAMB with 1% CAR (base + 1% VAR) nearly overlapped with that of the base bitumen. Therefore, a small amount of CAR (1%) cannot improve the fluidity of base bitumen and has no contribution to the compactability of the CAMB concrete. With the increase in CAR dosage, the bitumen viscosity experienced a rapid decline. Adding 3% CAR resulted in the bitumen viscosity decreasing from 155.5 mPa·s to 139 mPa·s at 165 °C, and from 202.5 mPa·s to 177.5 mPa·s at 145 °C. This could be attributed to the surface-active components in CAR which increase the lubricity of bitumen [26,30]. Therefore, the CAR is able to improve the fluidity and decrease the construction temperature of the base bitumen, which in turn has a positive effect on the blending efficiency and compaction performance of the bituminous mixture.

### 3.3. Pavement Performance of CAMB Concrete

#### 3.3.1. Anti-Rutting Property of CAMB Concrete

**Figure 7:**
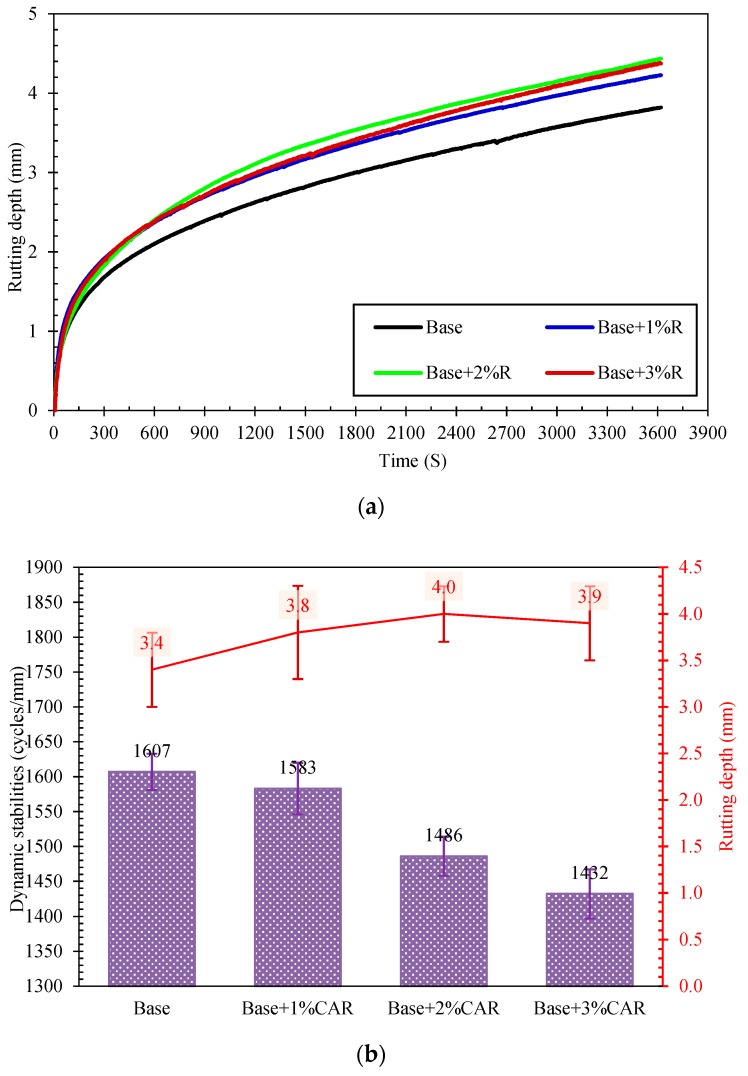
Influence of CAR on the rutting development of asphalt mixture: (**a**) rutting depth curve; (**b**) dynamic stability and rutting depth.

The rutting depth of the CAMB concrete versus loading cycles is shown in Figure 7. For all specimens, the rutting depth of asphalt slabs had an increasing trend with the prolonging of repeated tracking. It was found that the bituminous mixture without CAR had the lowest rutting depth of 3.4 mm. With the addition of the composite additive, its rutting depth experienced an obvious increase up to 4.0 mm. The dynamic stabilities of all mixtures are listed in Figure 7. The bituminous mixture without CAR had the highest dynamic stability, while it decreased for the CAMB mixtures. In detail, when compared with the bituminous mixture without CAR, the dynamic stability of the bituminous concrete with 1%, 2%, and 3% of CAR decreased by 1.5%, 7.5%, and 10.9%, respectively. This means that the CAR slightly reduced the dynamic stability of bituminous mixture. The decreased dynamic stability demonstrated a reduction in terms of the rutting resistance of the bituminous mixture, which may further deteriorate the performance of the corresponding pavements at high in-service temperatures.

This result is in contrast with the PI result of bitumen in Section 3.1 which showed increased rutting resistance potential after adding composite additive. The reason is that the rutting resistance of bituminous concrete is mainly determined by the aggregate skeleton and the interfacial friction between aggregate and bitumen. Due to the presence of surfactants in the CAR, the addition of the CAR can reduce the interfacial friction between aggregate and bitumen, which in turn causes the flow and slip of aggregates under repeated loading. Therefore, the dynamic stability of CAMB concrete tends to be lower than normal bituminous concrete, thereby reducing the rutting resistance. However, for the CAMB, the CAR can construct a solid form (especially when the temperature is lower than 60 °C) in the bitumen, and this phenomenon makes the bitumen stiffer and harder, which decreases its temperature sensitivity. Even though the dynamic stability of CAMB concrete decreased upon adding CAR, the deduction was not significant, as the dynamic stability of CAMB concrete with 3% CAR still satisfied the requirement 1000 cycles/mm.

#### 3.3.2. Low-Temperature Crack Resistance of CAMB Concrete

**Figure 8:**
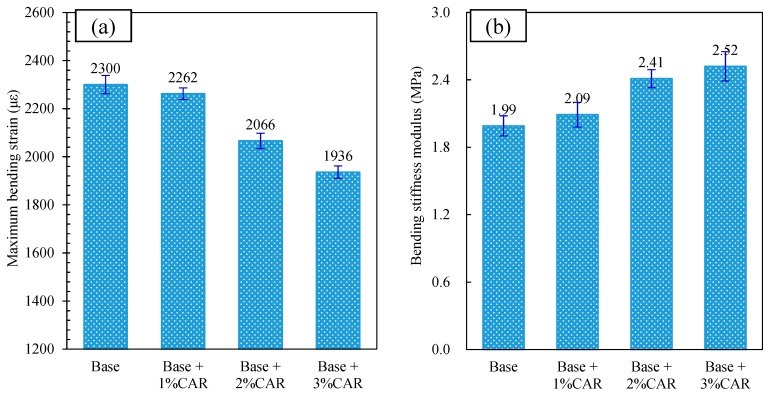
Influence of CAR on low-temperature crack resistance of bituminous concrete: (**a**) maximum bending strain; (**b**) bending stiffness.

The bending strain and bending stiffness of CAMB concrete are shown in Figure 8a. As shown in this figure, the bituminous concrete without CAR had the highest bending strain. By adding CAR, the bending strain of CAMB concrete experienced a rapid decrease and finally reduced by 15.8% after incorporating 3% CAR. The higher the bending strain is, the better the flexibility and crack resistance of CAMB concrete will be in a low-temperature situation. Based on the Specification for Design of Highway Asphalt Pavement (JTG D50-2017), to sustain low-temperature attack, the maximum bending strain of bituminous concrete in a cold winter zone should be over 2000 με. The CAMB concrete with 3% CAR cannot satisfy the minimum requirement for anti-cracking performance (see Figure 8b). The bending stiffness increased from 1.99 MPa to over 2.52 MPa with the increase in CAR dosage. A higher bending stiffness indicates a higher stress in the asphalt mixture under loading and, thus, the mixture is more prone to cracking. In summary, the CAR can decrease the maximum bending strain and increase the bending stiffness of bituminous concrete, which could have a negative effect on the anti-cracking performance of the bituminous pavement under a cold environment. However, the bituminous concrete with a CAR dosage lower than 2% still satisfied the requirement of 2000 με.

**Figure 9:**
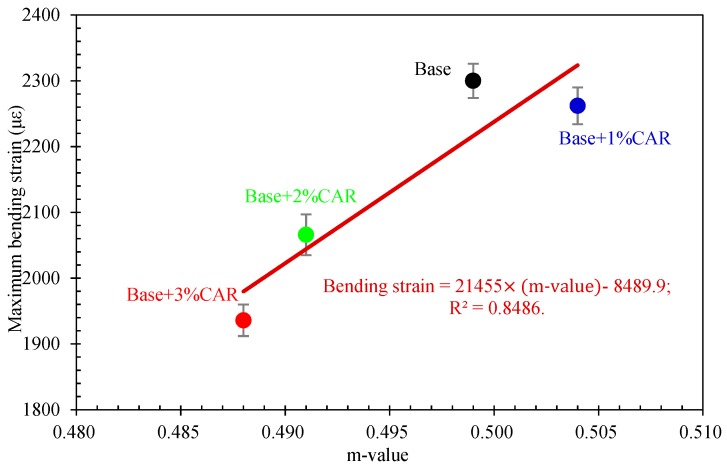
Relationship between m value of bitumen and bending strain of bituminous concrete.

**Figure 10:**
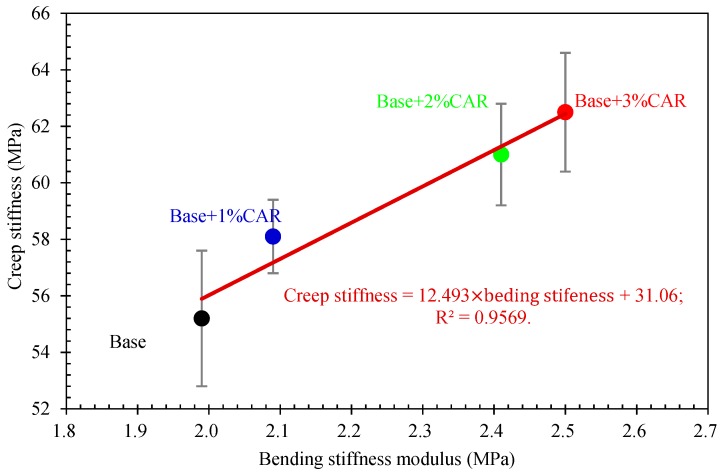
Relationship between creep stiffness of bitumen and bending stiffness of bituminous concrete.

The correlation between the anti-crack performance of bituminous binders and bituminous concretes was characterized by comparing related parameters. Figure 9 shows a linear relationship between the m value of bitumen and the bending strain of bituminous concrete. It was found that a greater m value corresponds to a greater maximum bending strain. Figure 10 further shows a linear relationship between the creep stiffness of bitumen and bending stiffness of bituminous concretes. The creep stiffness increased with the increase in bending stiffness. The results indicate that the BBR test and the 3PB test had good consistency in evaluating the anti-crack performance of the bituminous concretes. Moreover, the results agreed with the existing literature that the cracking resistance of asphalt mixture at low temperature is mainly controlled by the flexibility of the bituminous binder [32].

#### 3.3.3. Moisture Sensitivity of CAMB Concrete

**Figure 11:**
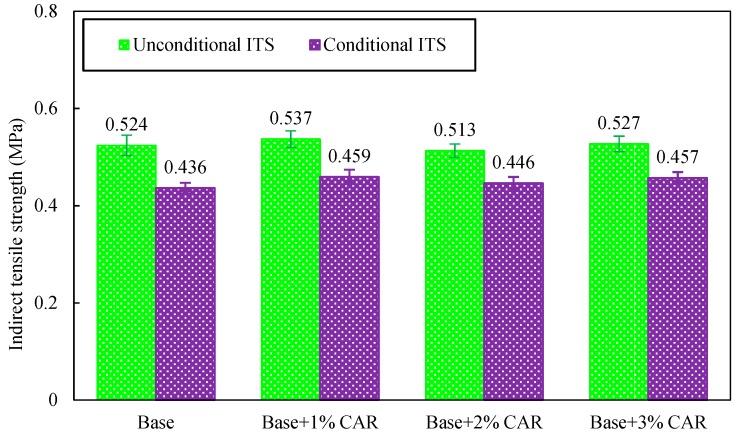
Indirect tensile strength (ITS) values of bituminous concrete before and after freeze–thaw cycle.

The ITS values of bituminous concrete before and after a freeze–thaw cycle are shown in Figure 11. As shown in this figure, there was no significant difference in the ITS value of bituminous concrete before the freeze–thaw cycle, no matter which dosage of CAR was incorporated. This phenomenon is consistent with previous findings [30]. Therefore, the CAR does not significantly influence the ITS of bituminous concrete before watering condition.

**Figure 12:**
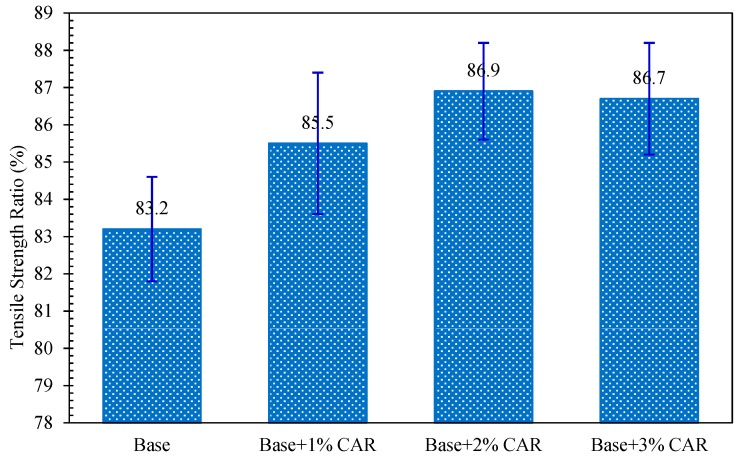
Tensile strength ratio (TSR) of bituminous concrete after freeze–thaw cycle.

On the other hand, the ITS values of all bituminous concrete had an obvious decrease during the freeze–thaw cycle. The residual ITS ratio of bituminous concrete after watering condition can be used to characterize its moisture sensitivity. The TSR was calculated according to Equation (6), and the results are presented in Figure 12. According to JTG D50-2017, in order to guarantee the moisture sensitivity of bituminous concrete being good enough to resist water damage, the TSR values should be no less than 75%. From Figure 12, the TSR value of bituminous concrete without CAR was 83.2%, which satisfies the moisture sensitivity requirement of 75%. After the addition of CAR, the TSR value of bituminous concrete experienced different levels of improvement, and the highest TSR value belonged to the specimen with 2% CAR dosage. This indicated that the moisture resistance of bituminous concrete with CAR is better than the bituminous concrete without CAR. The reason is that the cationic surfactants in the CAR can play the role of anti-stripping agents and improve the adhesion of the aggregate–bitumen interface, which enhances the resistance of mixtures to the moisture attack.

## 4. Conclusions

This study investigated the effect of a composite WMA additive (CAR) on the technical properties (physical and rheological performance) of bitumen and pavement properties (rutting, thermal crack, and moisture resistance) of bituminous concrete. The CAR contains both cationic surfactants and an organic additive. Based on the experimental results obtained in this research, the following conclusions can be obtained:

(1) Based on the physical tests of CAMB with a range of CAR content, the penetration of CAMB decreases, while the softening point and PI value increase, indicating that the CAMB tends to be stiffer and harder. This is because the solid form constructed by CAR in the bitumen can enhance the elasticity and high-temperature stability of bitumen, as well as decrease the temperature susceptibility.

(2) The rheological and viscosity teats of CAMB show that the CAR increases the creep stiffness and decreases the m value of bitumen, which means that CAR can reduce the low-temperature crack resistance of bituminous concrete. However, the deduction was not significant, as all CAMB samples were still in the same low-temperature PG grade as the bitumen without CAR. The obvious decreased viscosity of CAMB is due to the lower melting point of CAR, which leads to the better fluidity of bitumen, thereby improving its workability.

(3) The dynamic stability of CAMB concrete decreases with the addition of CAR, but the deduction is not significant; the dynamic stability of the CAMB concrete with 3% CAR still satisfied the requirement of 1000 cycles/mm.

(4) The CAR can decrease the maximum bending strain and increase the bending stiffness of bituminous concrete, which somehow could have a negative effect on the thermal cracking resistance of bituminous concrete. However, the CAMB concrete with CAR dosages lower than 2% still satisfied the requirement of 2000 με.

(5) CAR has no significant influence on the ITS of CAMB concrete before watering condition. However, after watering condition, CAR can increase the TSR of the CAMB concrete; therefore, it can enhance the moisture resistance of bituminous concrete.

## Figures and Tables

**Table 1:** Technical performance of the Pen 60/80 bitumen.

Technical Performance	Units	Results	Criteria	Methods
Penetration (100 g, 25 °C, 5 s)	0.1 mm	68	60–80	American Society for Testing and Materials (ASTM) D5
Softening point	°C	48.2	≥46	ASTM D36
Ductility (15 °C)	cm	150	≥100	ASTM D113

**Table 2:** Technical information of aggregates and filler of bituminous concrete.

Technical Information	Results			
	10–20 mm	5–10 mm	0–5 mm	Mineral Filler
Apparent specific gravity	2.738	2.725	2.734	2.674
Bulk specific gravity	2.724	2.671	2.674	-
Water absorption ratio (%)	0.4	0.7	0.8	-
Los Angeles abrasion loss (%)	19.3	-	-	-
Crushing value (%)	15.4	-	-	-
Sand equivalent (%)	-	-	69	-
Hydrophilic coefficient	-	-		0.51

**Table 3:** The physical and chemical information of Rediset.

Performance	Parameters	Description and Results
Physical performance	Color	Brown
	Bulk density (g/cc)	0.55
	Melting point (°C)	From 80 to 90
	Flash point (°C)	>150
Chemical performance	Solubility in water	Water-free
	Chemical composition	Long-chain aliphatic hydrocarbon and a $\;-{\mathrm{NH}}_{3}^{+}$ group

**Table 4:** Softening point (SP) and penetration results and the standard deviation (SD) of bitumen with CAR.

Property	Base		Base + 1% CAR		Base + 2% CAR		Base + 3% CAR	
	Result	SD	Result	SD	Result	SD	Result	SD
Softening point (°C)	48.2	0.2	48.4	0.2	49.8	0.3	51.5	0.2
Penetration (0.1 mm)	68.3	0.3	67.5	0.3	63.9	0.3	63.6	0.2

**Table 5:** Failure temperature of CAMB according to S and m values.

CAMB	Base		Base + 1% CAR		Base + 2% CAR		Base + 3% CAR	
	Result	SD	Result	SD	Result	SD	Result	SD
Failure temperature (°C)	−21.4	0.2	−21.3	0.2	−20.8	0.1	−20.6	0.2
